# Di­aqua­dichlorido­bis­(pyridine-κ*N*)cobalt(II)

**DOI:** 10.1107/S1600536813022484

**Published:** 2013-08-21

**Authors:** P.S. Kannan, A. S. Ganeshraja, K. Anbalagan, E. Govindan, A. SubbiahPandi

**Affiliations:** a Department of Physics, S. R. R. Engineering College (A. Jeppiaar Institution), Old Mamallapuram Road, Padur, Chennai 603 103, India; b Department of Chemistry, Pondicherry University, Pondicherry 605 014, India; c Department of Physics, Presidency College (Autonomous), Chennai 600 005, India

## Abstract

The title mol­ecule, [CoCl_2_(C_5_H_5_N)_2_(H_2_O)_2_], has -1 symmetry with the Co^II^ ion situated on an inversion centre. The cation has a distorted octa­hedral coordination environment and is surrounded by two N and two Cl atoms in the equatorial plane, while the coordinating water O atoms occupy the axial positions. The crystal exhibits nonmerohedral twinning with two domain states, the volume fractions of which were refined to 0.883 (2) and 0.117 (3). The crystal packing is stabilized by O—H⋯Cl hydrogen-bond inter­actions, forming two-dimensional networks lying parallel to (001). The crystal packing also features π–π inter­actions between the pyridine rings, with centroid–centroid separations of 3.493 (3) and 3.545 (3) Å.

## Related literature

For biological activity and potential applications of mixed-ligand cobalt complexes, see: Arslan *et al.* (2009 ▶) (anti­microbial activity); Delehanty *et al.* (2008 ▶) (anti­viral activity); Sayed *et al.* (1992 ▶) (anti­tumor activity); Teicher *et al.* (1990 ▶) (anti­tumor and cytotoxic activities); Milaeva *et al.* (2013 ▶) (biochemical properties of Co^II^). For related structures, see: Li *et al.* (2009 ▶); Zhu & Zhou (2008 ▶). For graph-set motifs, see: Etter *et al.* (1990 ▶).
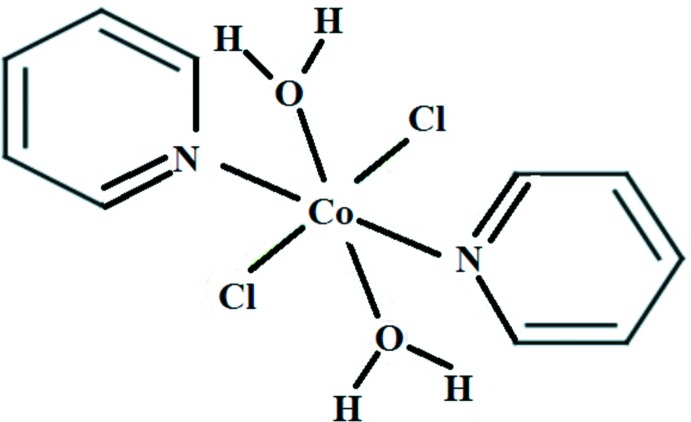


## Experimental

### 

#### Crystal data

[CoCl_2_(C_5_H_5_N)_2_(H_2_O)_2_]
*M* *_r_* = 324.06
Triclinic, 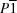

*a* = 6.2028 (2) Å
*b* = 6.5971 (1) Å
*c* = 8.5963 (2) Å
α = 109.734 (2)°
β = 102.621 (3)°
γ = 97.031 (2)°
*V* = 315.65 (1) Å^3^
*Z* = 1
Mo *K*α radiation
μ = 1.77 mm^−1^
*T* = 293 K
0.25 × 0.2 × 0.18 mm

#### Data collection

Oxford Diffraction Xcalibur diffractometer
Absorption correction: multi-scan (*CrysAlis PRO*; Oxford Diffraction, 2009 ▶) *T*_min_ = 0.576, *T*_max_ = 0.618
2211 measured reflections
2211 independent reflections
1926 reflections with *I* > 2σ(*I*)

#### Refinement

*R*[*F* ^2^ > 2σ(*F* ^2^)] = 0.047
*wR*(*F* ^2^) = 0.149
*S* = 1.16
2211 reflections
88 parameters
3 restraints
H atoms treated by a mixture of independent and constrained refinement
Δρ_max_ = 0.71 e Å^−3^
Δρ_min_ = −0.99 e Å^−3^

### 

Data collection: *CrysAlis CCD* (Oxford Diffraction, 2009 ▶); cell refinement: *CrysAlis RED* (Oxford Diffraction, 2009 ▶); data reduction: *CrysAlis RED*; program(s) used to solve structure: *SHELXS97* (Sheldrick, 2008 ▶); program(s) used to refine structure: *SHELXL97* (Sheldrick, 2008 ▶); molecular graphics: *ORTEP-3 for Windows* (Farrugia, 2012 ▶); software used to prepare material for publication: *SHELXL97* and *PLATON* (Spek, 2009 ▶) and *TwinRotMat* (Bolte, 2004 ▶).

## Supplementary Material

Crystal structure: contains datablock(s) global, I. DOI: 10.1107/S1600536813022484/fb2288sup1.cif

Structure factors: contains datablock(s) I. DOI: 10.1107/S1600536813022484/fb2288Isup2.hkl

Additional supplementary materials:  crystallographic information; 3D view; checkCIF report

## Figures and Tables

**Table 1:** Hydrogen-bond geometry (Å, °)

*D*—H⋯*A*	*D*—H	H⋯*A*	*D*⋯*A*	*D*—H⋯*A*
O1—H1*A*⋯Cl1^i^	0.82 (3)	2.45 (3)	3.266 (3)	176 (5)
O1—H1*B*⋯Cl1^ii^	0.81 (4)	2.41 (4)	3.156 (3)	153 (4)

Symmetry codes: (i) 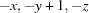; (ii) 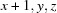.

**Fig. 1.**
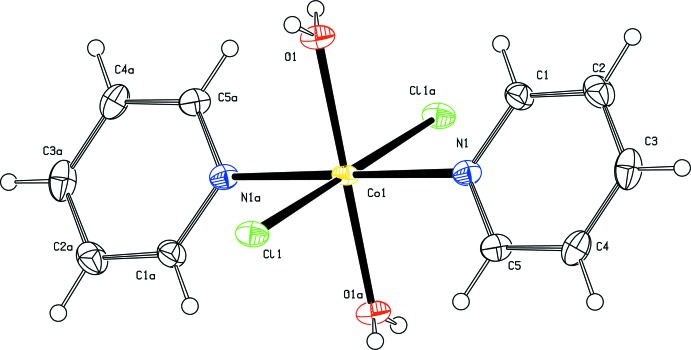
View of the title molecule with the atom labelling scheme. The displacement ellipsoids are drawn at the 30% probability level while the H atoms are shown as small spheres of arbitrary radii. The atoms labelled by "a" are related by the symmetry operation -x, -y, -z.

**Fig. 2.**
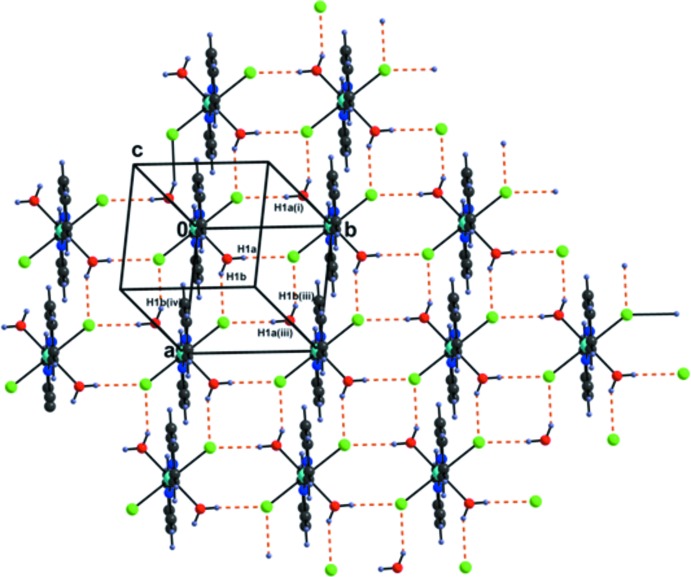
The hydrogen bond motifs in the title structure. Black: C; blue: N; light blue: Co; green: Cl, red: O; grey (small): H. Symmetry codes: (i): -x, 1 - y, -z, (iii): 1 - x, 1 - y, -z and (iv): 1 - x, -y, -z.

## supplementary crystallographic information

### 1. Comment

Mixed ligand cobalt complexes have found potential applications in the field of medicine because of its antitumor activity (Teicher *et al.*, 1990; Sayed *et al.*, 1992), antiviral activity (Delehanty *et al.*, 2008), antimicrobial activity (Arslan *et al.*, 2009) and radiosensitization and cytotoxic activities (Teicher *et al.*, 1990).

Cobalt is essential and integral component of vitamin B12, therefore it is found in many tissues. Cobalt complexes are useful in nutritional supplementation.

Electron transfer as well as ligand substitution reactions of cobalt(II) complexes are useful in understanding of biochemistry of cobalt(II) (Milaeva *et al.*, 2013). In order to study the electron transfer phenomena, the structure determination of the title compound, C_10_H_14_Cl_2_Co_1_N_2_O_2_, has been carried out.

The title molecule is shown in Fig. 1. It possesses symmetry 1 because its central atom Co(II) is situated at the crystallographic inversion centre. The Co(II) ion has a distorted octahedral coordination environment. It is surrounded by two N atoms and two Cl atoms in the equatorial plane, while the water oxygens occupy the axial positions.

The bond lengths are comparable with those observed in the related structure of tetraaquabis(pyridine-*kN*)cobalt(II) bis[4-amino-*N*- (6-chloropyridazin-3-yl)-benzenesulfonamidate] (Li *et al.*, 2009); tetraaquabis[5-(4-pyridyl)tetrazolido-*kN*^5^]cobalt(II) dihydrate (Zhu & Zhou, 2008). The crystal packing is stabilized by O1-H1A···Cl1 and O1-H1B···Cl1 hydrogen bonds with the graph-set motif *R*_2_^4^(8) (Etter *et al.*, 1990) with H1a and H1b and its equivalents generated by (iii): 1-*x*, 1-*y*, -*z*, and by two graph-set motifs *R*_2_^2^(8) with the atoms H1a and H1a^i^ or H1b and H1b^iv^ involved where (i): -*x*, 1-*y*, -*z* and (iv): 1-*x*, -*y*, -*z* (Table 1, Fig. 2).

There are also two π-electron ring···π-electron ring interactions between the pyridine rings N1//C1-C5 and the symmetry equivalents related by the operations (v): -*x*, *-y*, 1-*z* and (vi): -*x*, 1-*y*, 1-*z*. The respective distances between the centroids are 3.493 (3) and 3.545 (3)Å.

### 2. Experimental

Diaquadichloridobis(pyridine-N)Cobalt(II) complex was prepared by dissolving cobalt(II) chloride hexahydrate (CoCl_2_.6H_2_O, 1 g, 0.28 M) in boiling ethanol (C_2_H_5_OH, 15 ml). An excess of pyridine (C_5_H_5_N, 2.5 ml, 10 M) dissolved in ethanol (2.0 ml), was added slowly to this mixture in order to precipitate the title complex. The crude pink coloured precipitate was washed with cold ethanol and then air-dried. Then this precipitate was dissolved in 10-15 ml of hot ethanol, cooled down and allowed to crystallize. After cooling pink coloured crystals (0.84 g) developed within 12 hours. X-ray quality crystals were obtained by repeated recrystallization from hot ethanol. The typical size of the obtained block-like crystals was 0.8 × 0.6 × 0.5 mm. (The measured sample has been cut from a larger crystal.)

### 3. Refinement

After the solution of the phase problem by *SHELXS-*97 (Sheldrick, 2008), the refinement on *HKLF*4 (*SHELXL*-97, Sheldrick, 2008) converged to the *R*-factor equal to 0.067 for Fo>4σ(Fo). The difference electron density map showed several peaks of the order of magintude of 1eÅ^-3^. A check by *TwinRotMat* (Bolte, 2004; *PLATON* (Spek, 2009)) showed that the crystal had a two-fold non-merohedral twinning with the twin matrix [h_2_ k_2_ l_2_] = [h_1_ k_1_ l_1_][-1 0 0.731 /0 -1 0.964/0 0 1]. The twin law generated from |F_o_| - |F_c_| table was used to generate *HKLF*5 format file (Bolte, 2004) which is suitable for a twin refinement by *SHELXL*-97 (Sheldrick, 2008). The refinement converged to the *R*-factor of 0.0474 for Fo>4σ(Fo). All the spurious difference peaks have vanished. The refined domain fractions converged to the values 0.883 (2) and 0.117 (3). As the second component of the twin was weak it was not observed during the cell indexing.

All the hydrogens were discernible in the difference electron density map. Nevertheless all the aryl hydrogens were fully constrained. The values of the used constraints were following: C_aryl_H = 0.93 Å. *U*_iso_H=1.2*U*_eq_C_aryl_. The positional parameters of the water hydrogens were restrained with O-H = 0.82 (1)Å, while *U*_iso_(H_water-oxygen)_=1.5*U*_eq_(O_water_oxygen_).

### Crystal data

**Table:**

[CoCl_2_(C_5_H_5_N)_2_(H_2_O)_2_]	*Z* = 1
*M**_r_* = 324.06	*F*(000) = 165
Triclinic, *P*1	*D*_x_ = 1.705 Mg m^−^^3^
Hall symbol: -P 1	Mo *K*α radiation, λ = 0.71073 Å
*a* = 6.2028 (2) Å	Cell parameters from 2211 reflections
*b* = 6.5971 (1) Å	θ = 2.6–26.7°
*c* = 8.5963 (2) Å	µ = 1.77 mm^−^^1^
α = 109.734 (2)°	*T* = 293 K
β = 102.621 (3)°	Block, pink
γ = 97.031 (2)°	0.25 × 0.2 × 0.18 mm
*V* = 315.65 (1) Å^3^	

### Data collection

**Table:**

Oxford Diffraction Xcalibur diffractometer	2211 independent reflections
Radiation source: Fine-focus sealed tube, Enhance (Mo) X-ray Source	1926 reflections with *I* > 2σ(*I*)
Graphite monochromator	*R*_int_ = 0.000
ω scans	θ_max_ = 26.7°, θ_min_ = 2.6°
Absorption correction: multi-scan (*CrysAlis PRO*; Oxford Diffraction, 2009)	*h* = −7→7
*T*_min_ = 0.576, *T*_max_ = 0.618	*k* = −8→8
2211 measured reflections	*l* = −10→10

### Refinement

**Table:**

Refinement on *F*^2^	Primary atom site location: structure-invariant direct methods
Least-squares matrix: full	Secondary atom site location: difference Fourier map
*R*[*F*^2^ > 2σ(*F*^2^)] = 0.047	Hydrogen site location: difference Fourier map
*wR*(*F*^2^) = 0.149	H atoms treated by a mixture of independent and constrained refinement
*S* = 1.16	*w* = 1/[σ^2^(*F*_o_^2^) + (0.0878*P*)^2^ + 0.6139*P*] where *P* = (*F*_o_^2^ + 2*F*_c_^2^)/3
2211 reflections	(Δ/σ)_max_ < 0.001
88 parameters	Δρ_max_ = 0.71 e Å^−^^3^
3 restraints	Δρ_min_ = −0.99 e Å^−^^3^
22 constraints	

### Special details

**Table:**

Geometry. All esds (except the esd in the dihedral angle between two l.s. planes) are estimated using the full covariance matrix. The cell esds are taken into account individually in the estimation of esds in distances, angles and torsion angles; correlations between esds in cell parameters are only used when they are defined by crystal symmetry. An approximate (isotropic) treatment of cell esds is used for estimating esds involving l.s. planes.
Refinement. Refinement of F^2^ against ALL reflections. The weighted R-factor wR and goodness of fit S are based on F^2^, conventional R-factors R are based on F, with F set to zero for negative F^2^. The threshold expression of F^2^ > 2sigma(F^2^) is used only for calculating R-factors(gt) etc. and is not relevant to the choice of reflections for refinement. R-factors based on F^2^ are statistically about twice as large as those based on F, and R- factors based on ALL data will be even larger.

### Fractional atomic coordinates and isotropic or equivalent isotropic displacement parameters (Å^2^)

**Table:**

	*x*	*y*	*z*	*U*_iso_*/*U*_eq_	
C1	0.2308 (7)	0.2281 (6)	0.3845 (5)	0.0301 (9)	
H1	0.3593	0.2270	0.3453	0.036*	
C2	0.2576 (7)	0.3179 (7)	0.5588 (5)	0.0374 (10)	
H2	0.4012	0.3792	0.6352	0.045*	
C3	0.0695 (8)	0.3160 (7)	0.6187 (5)	0.0380 (10)	
H3	0.0834	0.3749	0.7361	0.046*	
C4	−0.1391 (7)	0.2252 (7)	0.5014 (5)	0.0339 (9)	
H4	−0.2690	0.2200	0.5386	0.041*	
C5	−0.1549 (7)	0.1418 (7)	0.3281 (5)	0.0297 (8)	
H5	−0.2975	0.0832	0.2499	0.036*	
N1	0.0267 (5)	0.1418 (5)	0.2680 (4)	0.0247 (7)	
O1	0.2699 (4)	0.2637 (4)	0.0415 (4)	0.0299 (7)	
H1A	0.264 (8)	0.388 (4)	0.045 (6)	0.045*	
H1B	0.365 (6)	0.210 (6)	0.000 (6)	0.045*	
Cl1	−0.27270 (14)	0.23327 (14)	−0.06014 (12)	0.0295 (3)	
Co1	0.0000	0.0000	0.0000	0.0209 (2)	

### Atomic displacement parameters (Å^2^)

**Table:**

	*U*^11^	*U*^22^	*U*^33^	*U*^12^	*U*^13^	*U*^23^
C1	0.028 (2)	0.028 (2)	0.029 (2)	0.0014 (16)	0.0024 (16)	0.0087 (17)
C2	0.040 (2)	0.029 (2)	0.031 (2)	0.0004 (18)	−0.0014 (19)	0.0059 (18)
C3	0.063 (3)	0.026 (2)	0.025 (2)	0.013 (2)	0.014 (2)	0.0074 (18)
C4	0.046 (3)	0.030 (2)	0.034 (2)	0.0138 (18)	0.0216 (19)	0.0147 (19)
C5	0.029 (2)	0.027 (2)	0.030 (2)	0.0039 (16)	0.0112 (16)	0.0053 (17)
N1	0.0250 (16)	0.0209 (15)	0.0256 (16)	0.0024 (12)	0.0082 (13)	0.0057 (13)
O1	0.0244 (15)	0.0231 (14)	0.0428 (17)	0.0016 (11)	0.0134 (13)	0.0117 (14)
Cl1	0.0237 (5)	0.0240 (5)	0.0383 (6)	0.0052 (4)	0.0082 (4)	0.0089 (4)
Co1	0.0170 (3)	0.0183 (4)	0.0228 (4)	−0.0001 (2)	0.0053 (3)	0.0035 (3)

### Geometric parameters (Å, º)

**Table:**

C1—N1	1.347 (5)	C5—H5	0.9300
C1—C2	1.376 (5)	N1—Co1	2.133 (3)
C1—H1	0.9300	O1—Co1	2.136 (2)
C2—C3	1.375 (6)	O1—H1A	0.817 (10)
C2—H2	0.9300	O1—H1B	0.812 (10)
C3—C4	1.372 (6)	Cl1—Co1	2.5078 (9)
C3—H3	0.9300	Co1—N1^i^	2.133 (3)
C4—C5	1.379 (6)	Co1—O1^i^	2.136 (2)
C4—H4	0.9300	Co1—Cl1^i^	2.5078 (9)
C5—N1	1.338 (5)		
			
N1—C1—C2	122.9 (4)	Co1—O1—H1A	129 (3)
N1—C1—H1	118.6	Co1—O1—H1B	108 (3)
C2—C1—H1	118.6	H1A—O1—H1B	115 (3)
C3—C2—C1	119.2 (4)	N1—Co1—N1^i^	180.0
C3—C2—H2	120.4	N1—Co1—O1^i^	92.24 (11)
C1—C2—H2	120.4	N1^i^—Co1—O1^i^	87.76 (11)
C4—C3—C2	118.5 (4)	N1—Co1—O1	87.76 (11)
C4—C3—H3	120.8	N1^i^—Co1—O1	92.24 (11)
C2—C3—H3	120.8	O1^i^—Co1—O1	180.0
C3—C4—C5	119.5 (4)	N1—Co1—Cl1^i^	89.65 (8)
C3—C4—H4	120.2	N1^i^—Co1—Cl1^i^	90.35 (8)
C5—C4—H4	120.2	O1^i^—Co1—Cl1^i^	88.46 (7)
N1—C5—C4	122.6 (4)	O1—Co1—Cl1^i^	91.54 (7)
N1—C5—H5	118.7	N1—Co1—Cl1	90.35 (8)
C4—C5—H5	118.7	N1^i^—Co1—Cl1	89.65 (8)
C5—N1—C1	117.3 (3)	O1^i^—Co1—Cl1	91.54 (7)
C5—N1—Co1	122.1 (3)	O1—Co1—Cl1	88.46 (7)
C1—N1—Co1	120.5 (3)	Cl1^i^—Co1—Cl1	180.0
			
N1—C1—C2—C3	1.5 (6)	C5—N1—Co1—O1^i^	−37.2 (3)
C1—C2—C3—C4	−0.4 (6)	C1—N1—Co1—O1^i^	140.2 (3)
C2—C3—C4—C5	−0.9 (6)	C5—N1—Co1—O1	142.8 (3)
C3—C4—C5—N1	1.2 (6)	C1—N1—Co1—O1	−39.8 (3)
C4—C5—N1—C1	−0.1 (6)	C5—N1—Co1—Cl1^i^	−125.6 (3)
C4—C5—N1—Co1	177.4 (3)	C1—N1—Co1—Cl1^i^	51.8 (3)
C2—C1—N1—C5	−1.3 (6)	C5—N1—Co1—Cl1	54.4 (3)
C2—C1—N1—Co1	−178.8 (3)	C1—N1—Co1—Cl1	−128.2 (3)

Symmetry code: (i) −*x*, −*y*, −*z*.

### Hydrogen-bond geometry (Å, º)

**Table:**

*D*—H···*A*	*D*—H	H···*A*	*D*···*A*	*D*—H···*A*
O1—H1*A*···Cl1^ii^	0.82 (3)	2.45 (3)	3.266 (3)	176 (5)
O1—H1*B*···Cl1^iii^	0.81 (4)	2.41 (4)	3.156 (3)	153 (4)

Symmetry codes: (ii) −*x*, −*y*+1, −*z*; (iii) *x*+1, *y*, *z*.
